# Electrical Resistivity Measurement with Spherical-Tipped Cylindrical Electrode Embedded on Two Layers

**DOI:** 10.3390/ma13092144

**Published:** 2020-05-06

**Authors:** Chang-Ho Hong, Song-Hun Chong, Gye-Chun Cho

**Affiliations:** 1 Division of Radioactive Waste Disposal Research, Korea Atomic Energy Research Institute (KAERI), Daejeon 34057, Korea; chhong@kaeri.re.kr; 2 Department of Civil Engineering, Sunchon National University, Jungang-ro 255, Sunchon 57922, Korea; shchong@scnu.ac.kr; 3 Department of Civil and Environmental Engineering, Korea Advanced Institute of Science and Technology (KAIST), Daejeon 34141, Korea

**Keywords:** electrical resistivity, layered medium, cylindrical electrodes with spherical tip, penetration depth, upper layer thickness, electrical resistivity mismatch

## Abstract

Complex geological processes form multiple layers and change pore water chemistry, saturation level, and temperature. Eventually, the strata hinder interpreting electrical resistivity data. There are no studies that theoretically explore the effects of electrode geometries and multiple layered systems on laboratory electrical resistivity measurements. This study formulates a theoretical electrical resistance between half spherical-tipped cylindrical electrodes embedded on two horizontal layers. The electrical resistivity of each layer is considered separately in the general electrical potential equation with different equipotential surface areas. The finite element analysis is conducted to validate the theoretical equation. Further interpretation provides insights into the distribution of electrical current flow under electrical resistivity mismatch for discussion.

## 1. Introduction

Electrical resistivity tests have been widely conducted at diverse scales, from a few centimeters for laboratory tests to kilometers for field tests. The measurement results allow for characterizing hydrochemical condition (e.g., the level of the groundwater table and the degree of soil contamination) and subsurface condition (e.g., soil profiling and anomaly detection) [1,2,3]. The field tests require enough distance among electrodes, yet laboratory-scale tests need to consider all of the geometries (radius and penetrated depth) of the electrodes, electrode spacing, and container size.

Strata have been formed by complex geological processes, including inevitable compositional variations with depth and unpredictable climate change, all of which ultimately lead to variations in pore water chemistry, saturation level, and temperature [4,5,6,7,8]. Previous studies employed (1) the method of image charges using the point electrode that fails to take into account electrode geometries or (2) Bessel function that cannot obtain analytical solution [9,10,11]. However, there is a need to theoretically explore the effects of electrode geometries and multiple layered system on laboratory electrical resistivity measurements.

This paper extends the previous study by authors of [12] to formulate a theoretical electrical resistance between half spherical-tipped cylindrical electrodes embedded on two horizontal layers. The electrical resistivity of each layer is considered separately in the general electrical potential equation with different equipotential surface areas. The finite element analysis is conducted to validate the theoretical equation. In addition, further interpretation explains the electrical current density distribution under electrical resistivity mismatch.

## 2. Theoretical Analysis

### 2.1. Homogeneous Ground (ρ_1_ = ρ_2_)

**Figure 1:**
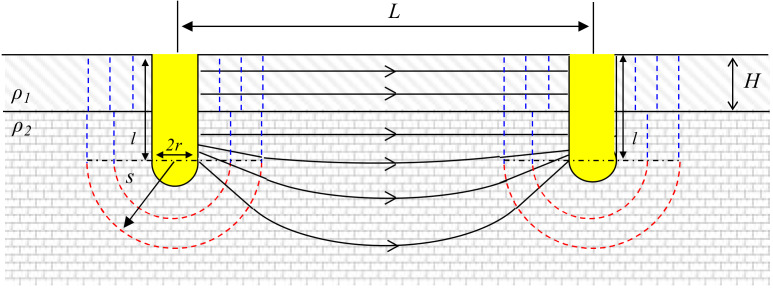
Definitions of the symbols used to represent two fully penetrated cylindrical electrodes embedded on two layers (*H* < *l*). *ρ_1_* is greater than *ρ_2_*.

Theoretical electrical resistance for two spherical-tipped cylindrical electrodes was formulated in a previous study [12]. In Figure 1, the same electrical resistivity of each layer (*ρ*_1_ = *ρ*_2_) indicates homogeneous medium. The equipotential surface area of a cylindrical electrode with half spherical end, whose radius is *r* and penetration depth is *l*, can be represented in terms of the shortest distance between the electrode center line and an arbitrary point *s*. As such, the equipotential surface area of one cylindrical electrode is given as

(1) $$A\left(s\right)=\underset{\mathrm{cylinder}\;\mathrm{side}}{\underbrace{2\pi sl}}+\underset{\mathrm{Half}\;\mathrm{sphere}}{\underbrace{2\pi s^{2}}}$$

The electric potentials from two cylindrical electrodes with rounded tip, separated by the length *L*, have the same quantities but opposite sign as the following equation when electric current *I* is the applied between two electrodes:

(2) $$V_{+}=-V_{-}=\int_{r}^{L-r}\frac{\rho I}{A\left(s\right)}ds=\frac{\rho I}{2\pi l}\left[\ln\left(1+\frac{l}{r}\right)-\ln\left(1+\frac{l}{L-r}\right)\right]$$

where *V_+_* is the potential from the positively charged electrode, *V_−_* is the potential from the negatively charged electrode, *ρ* is the electrical resistivity of the material, *I* is the applied electric current between two electrodes, and *L* is the distance between two cylindrical electrodes.

The electrical resistance between two cylindrical electrodes with rounded tip (*R_cylindrical_*) in a homogeneous medium, with electrical resistivity *ρ*, is given as follows:

(3) $$R_{cylindrical}=\frac{V_{+}-V_{-}}{I}=\frac{\rho }{\pi l}\left[\ln\left(1+\frac{l}{r}\right)-\ln\left(1+\frac{l}{L-r}\right)\right]$$

where *ρ* is the electrical resistivity of the material, *l* is the penetration depth of the electrodes, *r* is the radius of the electrodes, and *L* is the distance between two electrodes.

### 2.2. Electrical Resistance between Fully Penetrated Electrodes (H < l)

In a two-layer system, the two cylindrical electrodes can be installed with three different locations depending on the penetration depth *l*. First, we assumed that an additional layer exists in the ground and the cylindrical electrodes (with the radius *r* and penetration depth *l*) are placed across the upper and lower layers that have different electrical resistivity values (*ρ*_1_ and *ρ*_2_) as shown in Figure 1. The thickness of the upper layer *H* in this setup is smaller than the penetration depth of the electrodes *l*. The electrical resistivity mismatch among two layers has significant effects on equipotential surface shape and current flow magnitude. Assuming that the electric current flows in parallel similar to the parallel resistor system, the intensity gap can be considered separately in the general electrical potential equation with different equipotential surface areas. The electric potential of the positively and negatively charged cylindrical electrodes (*V*_1_ and *V*_2_) becomes

(4) $$V_{1}=-V_{2}=\int_{r}^{L-r}\frac{I}{\frac{A_{1}\left(s\right)}{\rho_{1}}+\frac{A_{2}\left(s\right)}{\rho_{2}}}ds$$

where *A*_1_(*s*) is surface area of upper layer (2π*Hs*) and *A*_2_(*s*) is surface area of lower layer [2π(*l* − *H*)*s* + 2π*s*^2^]. Therefore, the electrical resistance between two half spherical-tipped cylindrical electrodes which is partially buried in the bottom layer (*R*_1_) is

(5) $$R_{1}=\frac{V_{1}-V_{2}}{I}=\frac{\rho_{2}}{\pi \left(l-H+kH\right)}\ln\left(\frac{\left(L-r\right)\left(r+l-H+kH\right)}{r\left(L-r+l-H+kH\right)}\right)$$

where *k* is the electrical resistivity mismatch ratio between two layers (*k* = *ρ*_2_/*ρ*_1_). If the electrical resistivity of two layers becomes identical (i.e., *k* = 1), *R_1_* becomes identical to *R_cylindrical_* in Equation (3).

### 2.3. Electrical Resistance between Partially Penetrated Electrodes on Bottom Layer (l < H < l + r)

**Figure 2:**
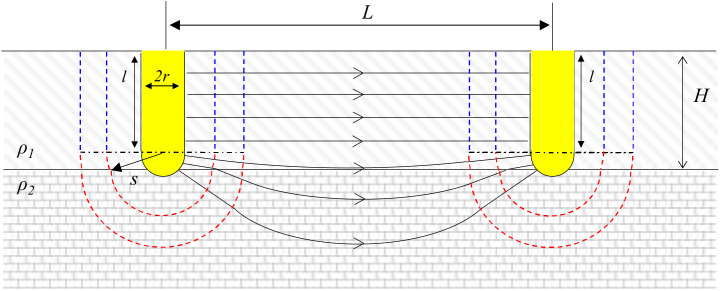
Definitions of the symbols used to represent two partially penetrated cylindrical electrodes embedded on two layers (*l* < *H* < *l* + *r*).

If the tip of the cylindrical electrodes is partially penetrated until the lower layer as in Figure 2, the current flow is distorted near the boundary of layer. In this case, the thickness of the upper layer (*H*) is smaller than the penetration depth of the electrodes (*l*).

Then, the electric potential of the positively and negatively charged cylindrical electrodes (*V*_3_ and *V*_4_) becomes

(6) $$V_{3}=-V_{4}=\int_{r}^{L-r}\frac{I}{\frac{A_{3}\left(s\right)-A_{4}\left(s\right)}{\rho_{1}}+\frac{A_{4}\left(s\right)}{\rho_{2}}}ds$$

where *A*_3_(*s*) is 2π*ls* + 2π*s*^2^, and *A*_4_(*s*) is 2πs(s + *l* − *H*). Therefore, the electrical resistance between two cylindrical electrodes which are partially buried in the bottom layer (*R*_2_) is

(7) $$R_{2}=\frac{V_{3}-V_{4}}{I}=\frac{\rho_{2}}{\pi \left(l-H+kH\right)}\ln\left(\frac{\left(L-r\right)\left(r+l-H+kH\right)}{r\left(L-r+l-H+kH\right)}\right).$$

### 2.4. Electrical Resistance between Electrodes Penetrated Only into Upper Layer (l < H − r)

**Figure 3:**
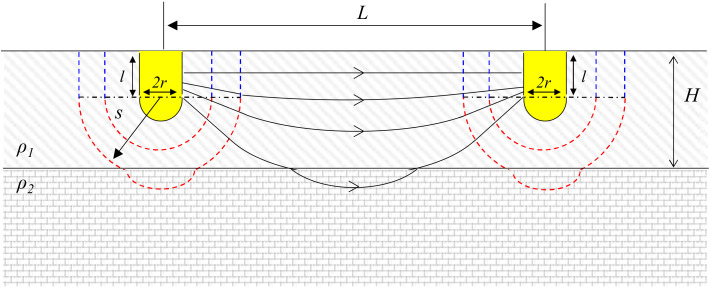
Definitions of the symbols used to represent cylindrical electrodes partially penetrating the upper layer of a two-layered ground media.

Two electrodes penetrated only into the upper layer, yet the current flow initiated from the upper layer is slightly affected by the lower layer (Figure 3). The electric potential of cylindrical electrodes consists of two parts: The first part considers the equipotential surface until boundary between two layers. In the second part, the equipotential surface starts from the boundary until opposite electrode surface, yet the electrical potential equation should remove the equipotential surface of lower layer *A*_4_(*s*):

(8) $$V_{5}=-V_{6}=\int_{0}^{H-l}\frac{\rho_{1}I}{A_{3}\left(s\right)}ds+\int_{H-l}^{L-r}\frac{I}{\frac{A_{3}\left(s\right)-A_{4}\left(s\right)}{\rho_{1}}+\frac{A_{4}\left(s\right)}{\rho_{2}}}ds$$

The electrical resistance between two cylindrical electrodes which is partially buried in the upper layer (*R*_3_) is

(9) $$R_{3}=\frac{V_{5}-V_{6}}{I}=\frac{\rho_{1}}{\pi l}\ln\left(\frac{\left(H-l\right)\left(r+l\right)}{Hr}\right)+\frac{\rho_{2}}{\pi \left(l-H+kH\right)}\ln\left(\frac{kH\left(L-r\right)}{\left(L-r+l-H+kH\right)\left(H-l\right)}\right).$$

## 3. Finite Element Simulation

### Numerical Simulations in a Large Container

**Figure 4:**
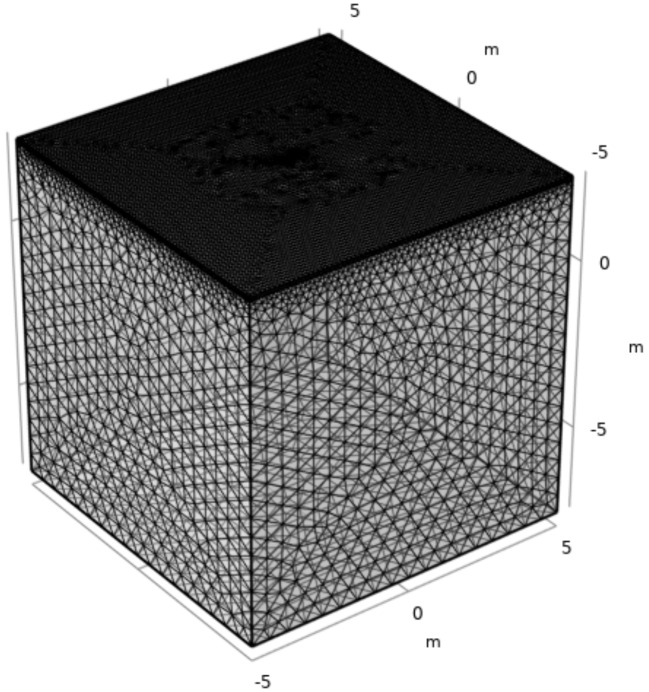
Tetrahedral mesh generated in COMSOL Multiphysics.

Finite element analysis using COMSOL Multiphysics was conducted to validate the derived equations. The AC/DC module solves partial differential equations (Laplace’s equation) and represents the electrical density distribution among electrodes. As shown in Figure 4, tetrahedral meshes are constructed using the inherent mesh generation function in the numerical simulation tool wherein the finer mesh is generated near two electrodes. The model geometry corresponds to a 10-m cube. The side and bottom boundary were modeled with an insulation layer. The electrode is located 4 m away from size boundary and, thus, the boundary effect becomes negligible. A 1−A electric current was applied to one electrode and the electrical resistance between the two electrodes was obtained using the potential difference divided by the applied electric current. Forty-eight cases were simulated to see the smooth trend line compared to theoretical equation which varies with the distance between the two electrodes (*L*), the penetration depth (*l*), the radius (*r*), the thickness of the upper layer (*H*), and the electrical resistivity ratio (*k*). All cases are listed in Table 1.

**Figure 5:**
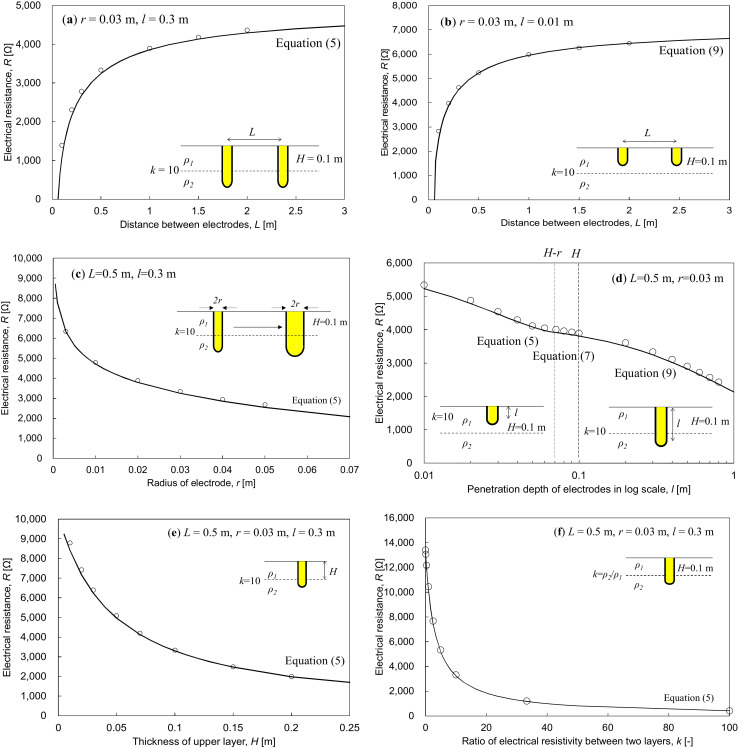
Variation of electrical resistance with respect to electrode geometries embedded on two layers: (**a**) and (**b**) electrode distance between two cylindrical electrodes *L*; (**c**) radius *r* when *l* = 0.3 m; (**d**) penetration depth when *H* = 0.1 m, (**e**) the thickness of upper layer when *l* = 0.3 m; (**f**) electrical resistivity ratio when *l* = 0.3 m. Solid lines are obtained from Equations (5), (7), and (9), and the hollow dots are obtained using numerical simulation.

Figure 5 presents the theoretical and numerical resistances as a function of electrode geometries embedded on two layers. In all cases, the numerical simulation results show good agreement with the corresponding theoretical equation. For both fully and partially penetrated cases, the electrical resistance rapidly increased with electrode spacing and the increment was eventually reduced. The fully penetrated case produced relatively lower electrical resistance (Figure 5a,b). Note that while the electrode is embedded on only the upper layer with lower electrical resistivity and the electrical resistance increased due to smaller contact area of the electrode. Larger electrode radius and deeper penetration depth decrease electrical resistance (Figure 5c,d). In particular, a transitional zone (little variation of electrical resistance) exists as the electrode penetrates into lower layer. After the penetration depth becomes greater than the thickness of the upper layer (full penetration), the electrical resistance is rapidly reduced. Furthermore, as the bottom layer was replaced by upper layer (higher resistivity), the electrical resistance decreased (Figure 5e). Note that if the electrical mismatch ratio is less than 1 (upper layer has lower resistivity), the electrical resistance will increase. Lastly, as the electrical mismatch ratio increased with the fully penetrated case, the electrical resistance dramatically decreased (Figure 5f).

## 4. Discussion

**Figure 6:**
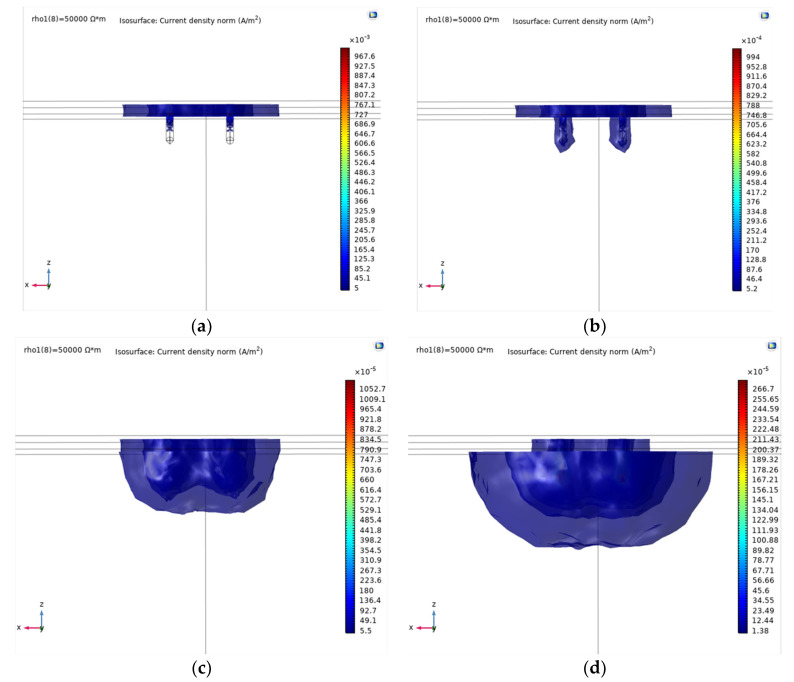
Electrical current density in different electrical mismatch ratio: (**a**) *k* = 100; (**b**) *k* = 10; (**c**) *k* = 1; (**d**) *k* = 0.1. The electrode geometries are radius *r* = 0.03 m, penetration depth *l* = 0.3 m, and distance *L* = 0.5 m. The layer formations are thickness of upper layer *H* = 0.1 m and the bottom layer’s electrical resistivity *ρ*_2_ = 5000 Ω∙m.

Numerical results are further analyzed to investigate how electrical current flows through the two-layer system. Figure 6 presents the current density distribution under different electrical resistivity mismatch ratio. Note that the current density is directly related to the electrical resistivity of each layer by Ohm’s law (*J* = *E*/*ρ*). Isotropic surface of the current density is obviously separated between upper and lower layer when *k* is not unity (k ≠ 1). When the electrical resistivity mismatch ratio is greater than 1 (*k* = 10 and 100), the electrical current mostly flows upper layer with lower electrical resistivity. Higher *k* decreases current density distribution, and eventually concentrates the current flow on upper layer. In unity *k*, there is no distortion of current density contour at the boundary between two layers. Lower *k* widely spreads the current flow from the boundary to the bottom layer.

## 5. Conclusions

The theoretical electrical resistance between two spherical-tipped cylindrical electrodes embedded on two layers was derived for layered media using the equipotential surface area considering the electrical resistivity of each layer. Numerical simulations were performed using finite element analysis (COMSOL Multiphysics) to verify the theoretical equations. The main conclusions of the study are as follows:

1. The derived equations for the electrical resistance between two spherical-tipped cylindrical electrodes with different penetration depths can be represented as a combination of the inherent electrical resistivity of two layers (*ρ*_1_ and *ρ*_2_), the thickness of the upper layer (*H*), and the electrode geometries (i.e., the radius (*r*) and penetration depth (*l*)) and the distance (*L*) between them.
2. The numerical simulations verified the derived equations. The electrical resistance between two cylindrical electrodes with rounded tips were obtained from the numerical simulations under various combinations of electrode distance (*L*), radius (*r*), and penetration depth (*l*), the thickness of the upper layer (*H*), and the electrical resistivity mismatch ratio between the upper and lower layers (*k*). The theoretical and simulated electrical resistance values were well matched.
3. The contours of the current density which are directly related to the electrical resistivity of each layer by Ohm’s law are obtained in different electrical resistivity mismatch ratio (*k*). The resistivity mismatch ratio between two layers significantly affect how the electric current flows. The electric current tends to flow through the less resistive layer.

The theoretical electrical resistances evolve with electrode geometries and layer formation history (thickness of upper layer and electrical resistivity mismatch ratio). However, it still remains difficult to characterize subsurface conditions because there are more unknowns than number of resistivity measurements due to ground heterogeneity. Thus, there is a need to investigate the uniqueness of solution for electrical resistivity survey. The back-analysis results can optimize the electrode geometries and electrical resistivity distribution.

## Figures and Tables

**Table 1:** Parametric studies by varying electrode geometries and electrical resistivity mismatch ratio for the numerical simulations (a total of 48 cases are selected).

Distance *L* (m)	Penetration Depth *l* (m)	Radius *r* (m)	Thickness of Upper Layer *H* (m)	Electrical Resistivity Mismatch Ratio *k* = *ρ_2_*/*ρ_1_*
7 Cases	11 Cases	6 Cases	13 Cases	9 Cases
0.1 0.2 0.3 0.5~2 (in each 0.5 m)	0.01~0.09 (in each 0.01 m) 0.1~0.8 (in each 0.1 m)	0.003 0.01~0.05 (in each 0.01 m)	0.01 0.02 0.03 0.05 0.07 0.1 0.15 0.2~0.7 (in each 0.1 m)	100 33.3 10 5 2.5 1 0.33 0.1 0.025
